# Influence of different infill materials on the performance of geocell-reinforced cohesive soil beds

**DOI:** 10.1038/s41598-023-39580-x

**Published:** 2023-07-30

**Authors:** Yang Zhao, Zheng Lu, Jie Liu, Jingbo Zhang, Hailin Yao

**Affiliations:** 1 State Key Laboratory of Geomechanics and Geotechnical Engineering, Institute of Rock and Soil Mechanics, Chinese Academy of Sciences, Wuhan, 430071 China; 2 Hubei Key Laboratory of Geo-Environmental Engineering, Wuhan, 430071 China; 3 Xinjiang Transportation Planning Survey and Design Institute Co., Ltd, Urumqi, 830006 China; 4 CCCC Second Highway Consultants Co., Ltd., Wuhan, 430056 China

**Keywords:** Planetary science, Solid Earth sciences

## Abstract

This paper presents a comprehensive study on the numerical and parametric study of geocell-reinforced cohesive soil beds, focusing on different infill materials. The numerical calculations were validated against model test results using FLAC^3D^ software. Subsequently, the verified model was expanded to the geocell-reinforced cohesive soil beds. Six cases were simulated to investigate the reinforced performance, including pressure-settlement responses, bearing capacity improvement factor, settlement reduction percentage, and surface deformation. The numerical findings emphasize that the significance of superior geocell reinforcement should not overshadow the consideration of soil infill’s mechanical properties. In the case of cohesive soil as the infill material, the poor improvement in geocell-reinforced performance may be attributed to its low modulus and cohesion. Parametric studies suggest that geocells significantly impact reinforced performance when the infill material consists of foundation soil with a higher modulus and lower cohesion. Further, according to this numerical study, cohesionless soil with a modulus of 20 MPa and friction of 40° is the optimum infill soil in pockets to reinforce cohesive soil beds.

## Introduction

Geocells have a foldable and honeycomb-shaped geometry, which can improve the apparent cohesion of the soil due to the three-dimensional lateral limitation (LL) system. The pockets of the geocell structure are filled with granular materials, which are then compacted to create a reinforced composite layer. Due to the excellent reinforced performance and economical, geocells have been widely applied in geotechnical engineering^1–8^. Geocells increase soil cohesion while maintaining friction by providing LL through their vertical walls. In addition to the LL effect provided by geocells, two other reinforced effects are observed under static loading: vertical stress dispersion and the membrane mechanism^9^. Furthermore, geocells can isolate vibrations and decrease dynamic stress under dynamic loads^9–12^.

Model plate load tests are widely used to assess geocell-reinforced soil beds' bearing capacity. Dash et al.^13,14^ conducted a laboratory-model test to study the improvement of the bearing capacity of strip footings supported on geocell-reinforced sand regarding pressure-settlement curves, bearing capacity improvement factors, and surface settlement/heave. By analyzing some parameters, including the geocell size and modulus, depth of the geocell mattress, and the relative density of the sand, the author claimed that the top of the geocell mattress should be at a depth of 0.1-time footing width to obtain the maximum reinforced performance. Following this research, subsequent studies by Ujjawal et al.^11^, Hegde and Sitharam^15^, Hegde and Sitharam^16^, Hegde and Sitharam^17^, Hegde and Sitharam^18^, Venkateswarlu et al.^19^ all adopted this buried depth of geocell mattress to study the behavior of reinforced soil beds based on model or site tests. Historically, researchers have primarily focused on enhancing the bearing capacity of geocell-reinforced beds^20,21^, load distribution of geocell mattresses^22^, and vibration isolation^11,12^. These research findings have greatly influenced the application of geocells in geotechnical and subgrade engineering. Furthermore, concerning numerical technology, it has been accepted by many researchers to study the behavior of geocell-reinforced soil beds. Ujjawal et al.^11^, Hegde and Sitharam^23^, Latha and Somwanshi^24^ employed the equivalent composite approach (ECA) to simulate the geocell-soil composite layer. However, as modeling techniques advance, using actual 3D models to simulate geocell-soil interaction has become more prominent. Han et al.^25^ and Latha and Somwanshi^24^ adopted the diamond pattern to simulate the geocell shape. Further, Leshchinsky and Ling^26^, Biabani et al.^27^, Ngo et al.^28^, Siabil et al.^29^ used the square and hexagon pattern to calculate. The honeycomb shape (actual shape) was also adopted in recent years^17,19,30^. Overall, employing the actual shape of geocells in numerical models can accurately represent the behavior of geocell-reinforced soil beds, including pressure-settlement response and surface settlement/heave. Numerical software enables efficient calculation of various cases by adjusting parameters, allowing for direct visualization of reinforced mechanisms and stress distributions through displacement and stress contours.

Regarding the infill materials used in geocell pockets, cohesionless soil is predominantly utilized in geocell-reinforced engineering for both geocell-reinforced sand and cohesive soil beds^3,20,22,31–33^. The experimental results of Biswas et al.^34^ proved that the soil infill was the critical parameter that affected the reinforced performance. Also, Sireesh et al.^32^ claimed that the geocells filled with dense soil were beneficial to improving bearing capacity. Hegde and Sitharam^35^ compared the performance of three infill materials: local red soil, sand, and aggregate. The bearing capacity of geocell-reinforced beds increased by thirteen times for aggregate infill, eleven times for sand infill, and ten times for red soil infill, indicating the minimal influence of infill materials on geocell performance. In fact, the mechanical properties of the soil infill, rather than the specific type of soil, play a crucial role. In addition to sand, various other materials such as silty sand, slag, aggregate, soft soil, clay, rubber-soil mixtures, and recycled asphalt pavement materials have been used as infill materials in model tests conducted by Sitharam and Sireesh^36^, Thallak et al.^37^, Krishnaswamy et al.^38^, Thakur et al.^39^, Mehrjardi et al.^40^, Pokharel^41^, Venkateswarlu and Hegde^42^. These research results demonstrate that both cohesive and non-cohesive soils can serve as suitable infill materials for geocell-reinforced soil beds, offering excellent reinforcement performance. According to Bahadir et al.^43^, construction and demolition materials can also be considered as alternative infill materials to virgin aggregates. In the case of geocell-reinforced cohesive soil beds, typically, three options for soil infill are available: (1) cohesionless soil, (2) cohesive soil with superior mechanical properties, and (3) cohesive soil identical to the existing soil beds. Option 1 and Option 2 enhance the performance of reinforced soil beds, while Option 3 potentially reduces transportation costs as there is no need to bring soil from other areas. However, considering these three options, limited research has been conducted on the influence of modulus and shear strength of soil infill on reinforced performance. The experimental results from Bahadir et al.^43^ also suggested that construction and demolition materials also can be used as an alternative infill material to virgin aggregates. Regarding the geocell-reinforced cohesive soil beds, there are usually three options of soil infill to be selected: (1) cohesionless soil; (2) cohesive soil with superior mechanical properties; (3) cohesive soil as the same as soil beds. Option 1 and Option 2 can benefit the performance of reinforced soil beds, while Option 3 probably saves many costs, that is, the persons do not need to transport the soil from other areas. However, based on the three options, few researchers studied the influence of modulus and shear strength of soil infill on the reinforced performance.

The study aimed to examine the performance of geocell-reinforced cohesive soil beds with various infill materials to maximize the soil infill contribution and determine suitable mechanical parameters. Initially, the geocell-reinforced soil beds were modeled using the FLAC^3D^ explicit finite difference package, and the results were compared with those obtained from a laboratory model test referenced in the literature. Subsequently, the validated model was extended to geocell-reinforced cohesive soil beds to analyze the impact of the mechanical parameters on the bearing capacity. The analysis included pressure-settlement responses, the bearing capacity improvement factor ($I_{f}$), and the percentage reduction in settlement (PRS). As mentioned earlier, numerical studies were performed using Option 1 and Option 3 to simulate real subgrade or foundation engineering scenarios more effectively. Notably, a sand/aggregate cushion was also utilized to ascertain the proportion of geocell's contribution to the reinforcement. Lastly, a parameter study was conducted to determine the appropriate mechanical parameters for the soil infill.

## Validation of numerical calculation

**Figure 1:**
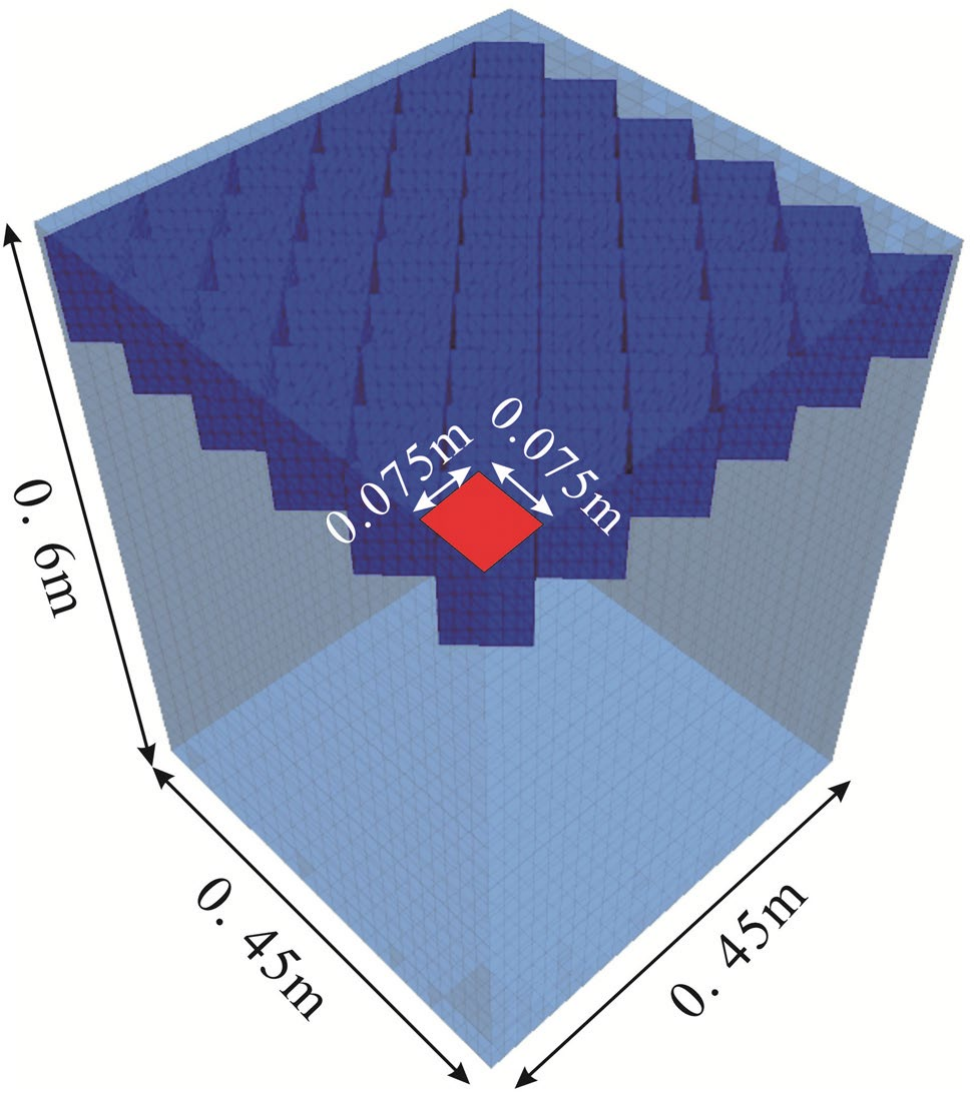
Geometry of FLAC^3D^ model for validation analysis.

The validation of geocell-reinforced models was performed by simulating the model tests by Latha and Somwanshi^24^, and the pressure-settlement response of the models was compared with the experimental data. Latha and Somwanshi^24^ performed laboratory model loading tests on square footings (25 mm thick and size of 150 × 150 mm) supported on geocell-reinforced sand beds. The geocell used had a diamond pattern and was constructed using biaxial geogrid (polypropylene) and geonet (high-density polyethylene). The equivalent composite approach (ECA) was adopted in the numerical simulations to compare the results with those obtained experimentally. However, in recent years, researchers have increasingly considered models that replicate the actual shape of geocells. Therefore, this study utilized the geogrid structure element available in FLAC^3D^ to simulate the diamond pattern geocells constructed using biaxial geogrids. The geocell-reinforced layer was prepared using the following combination of parameters: $u/B = 0.05$, $d/B = 0.55$, $b/B = 6$, $h/B = 0.6$ to match the experimental placement. Here, $u$, $d$, $b$, $h$, and $B$ represent the depth of placement of geocell layer, equivalent pocket diameter, width of the geocell mattress, geocell height, and width of footing, respectively. Notably, the geogrid structure element in FLAC^3D^ offers an essential mechanism known as the interface shear behavior. The interface shear relationship between the geocell and the infill materials was considered linear with the Mohr–Coulomb failure criterion^11^. The interface shear modulus parameter was calculated following the work of Yang et al.^44^. Additionally, the interface cohesion and friction were determined as per Oliaei and Kouzegaran^45^.

$${\text{Interface}}\,\,{\text{friction}}\,\,{\text{angle}}\,\, = \,\,{\text{atan }}\left( {0.8 \times \tan \left( \upvarphi \right)} \right).$$

$${\text{Interface}}\,\,{\text{cohesion}}\, = \,0.8 \times {\text{c}}{.}$$

where $\upvarphi$ and ${\text{c}}$ are the friction angle and cohesion strength of soil infill, respectively. In addition, it is acknowledged that in geocell-reinforced structures, the maximum strain of the geocell is less than 1% or 2%^46,47^. Hegde and Sitharam^16^ used the secant modulus corresponding to geocells' 2% axial strain to obtain the young modulus. Within such a small deformation range, the geocell can be considered elastic. Hence, the linear elastic and Mohr–Coulomb constitutive models were used to simulate the behavior of geocell and soil (including the infill materials and soil bed beneath the geocell-reinforced layer), respectively. The modulus of polypropylene was determined to be 1GPa through back calculation in this numerical simulation due to the excellent tensile property of polypropylene^48^. The soil properties were obtained from the studies of Latha and Somwanshi^24^. Specific values are provided in Table 1. Moreover, a quarter portion was modeled to reduce the computational effort. The quarter symmetric model of size was 0.45 m × 0.45 m × 0.6 m. It was noticed that the pressure-settlement response is related to the increased number of zones. The change in pressure-settlement curves was found negligible when the number of zones was beyond about 15,000. Hence, at last, the number of zones was considered 18,522 to simulate the unreinforced and geocell-reinforced soil beds. Figure 1 shows the view of the FLAC^3D^ model for the diamond pattern geocell-reinforces soils. The bottom displacement, representing the tank bottom, was constrained in all three directions, while the four side boundaries, symbolizing the tank sides, were restricted solely in the normal direction, allowing displacement in the vertical direction. To simulate the roughness of the footing, lateral resistance was applied to the grid points corresponding to the footing area. In the analysis, the loading area remained consistent with the model test, and controlled velocity loading of 1e−6 m/step was implemented. Vertical displacement was incrementally increased to induce a predetermined value of footing settlement.

**Table 1:** Properties of soil and geocell in the validation modeling.

Parameters	Value
Sand	
Young modulus, $M_{s}$ (MPa)	4.5
Poisson’s ratio, $\vartheta$	0.3
Cohesion, $c_{s}$ (kPa)	0
Friction, ${{\upvarphi }}_{s}$ (°)	44
Dilation, ${{ \varPsi }}_{s}$ (°)	29.3
Density, $\uprho$ (kg/m^3^)	2100
Geocells	
Young modulus, $M_{g}$ (MPa)	1000
Poisson's ratio, $\vartheta$	0.45
Interface shear modulus $k_{i}$ (MPa/m)	19.7
Interface cohesion, $c_{i}$ (kPa)	0
Interface friction, $\upvarphi_{i}$ (°)	37.7
Thickness of geocell, ${\text{t}}$ (mm)	1

**Figure 2:**
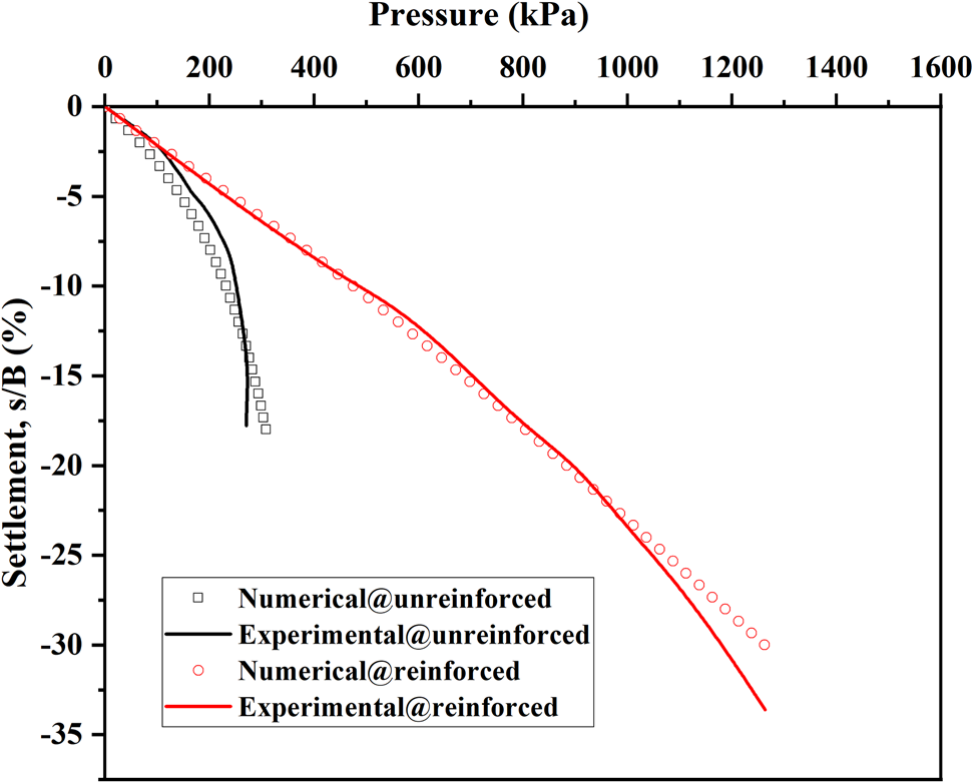
Pressure-settlement curves relationship of the validation and results.

Figure 2 shows the comparison of numerical results and experimental results. According to the figure, the four curves, including experimental and numerical results, almost overlap. Concerning the case of geocell reinforced, it can be concluded that using the actual geocell shape in simulation is accurate enough. And the pressure-settlement response under the case of unreinforced and geocell-reinforces can be successfully simulated in FLAC^3D^ models with or without the structure element simulating geocells. Gedela and Karpurapu^3^, Venkateswarlu and Hegde^49^ all also used FLAC^3D^ to simulate the geocell-reinforced soil beds.

## Numerical analysis of geocell-reinforced cohesive soil beds

In this study, the verified model shown in Section “Validation of numerical calculation” was extended to the geocell-reinforced cohesive soil beds model. This approach aligns with the methodology employed by Oliaei and Kouzegaran^45^. Only the honeycomb-shaped geocells and cohesive soil were changed to simulate the actual engineering located in a seasonally frozen area in Harbin, Heilongjiang province, China. The cohesive soil used in this study was taken from here. It is important to note that the model's dimensions, footing size, loading conditions, boundary conditions, constitutive model, and geocell-soil interface parameters remained consistent with the initial model to ensure a comprehensive and reliable analysis.

As outlined in the “Introduction” section, three options are typically available for selection. However, in this study, only Option 1 and Option 3 were chosen to examine and compare the impact of soil infill on the reinforced performance. Table 2 illustrates the scenarios for both the unreinforced and reinforced cases, considering various soil infill conditions within the geocell pockets. It is important to emphasize that the sand/aggregate cushion was also incorporated to quantify the proportion of the geocell's contribution to the overall reinforcement.

**Table 2:** Detailed information of unreinforced and reinforced case.

No	Name	Details	Characteristics
1	Unreinforced	–	–
2	GRCS	Geocell reinforced cohesive soil beds with cohesive soil as the infill materials	Cohesive soil infill in geocell pockets as the same as cohesive soil beds
3	SC	Sand cushion	As the contrast calculation compared to GRS and GRA cases, to investigate the percentage of geocell’s contribution
4	AC	Aggregate cushion	
5	GRS	Geocell-reinforced cohesive soil beds with sand as the infill materials	Comparing the influence of different cohesionless soil infill on the reinforced performance
6	GRA	Geocell-reinforced cohesive soil beds with aggregate as the infill materials	

**Figure 3:**
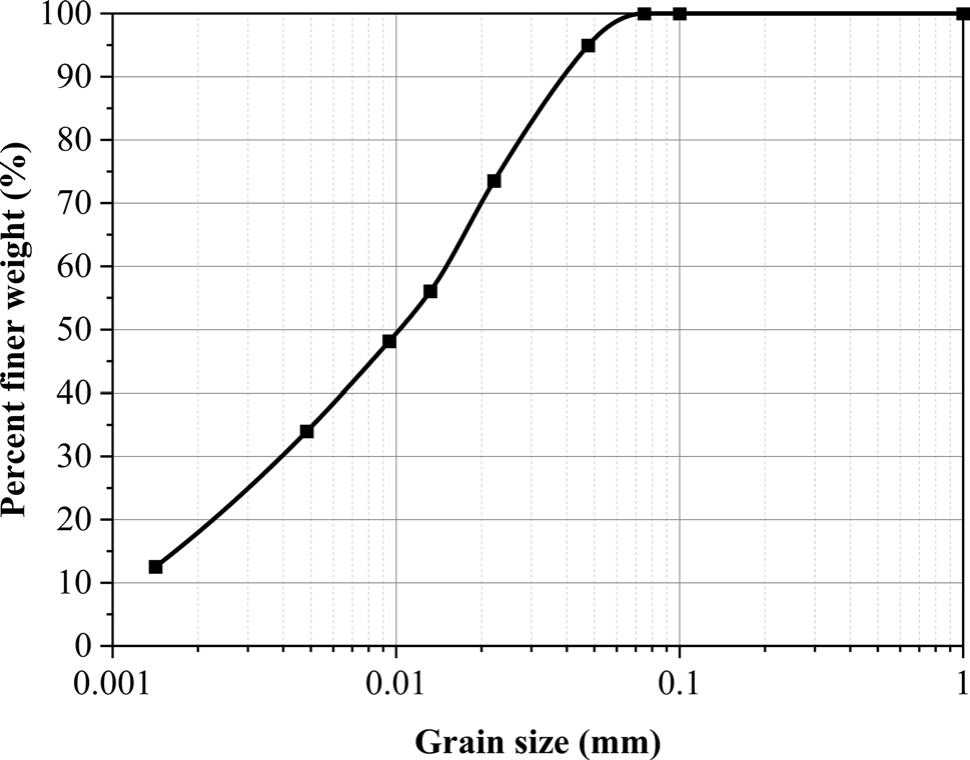
Particle size distribution of cohesive soil^50^.

**Figure 4:**
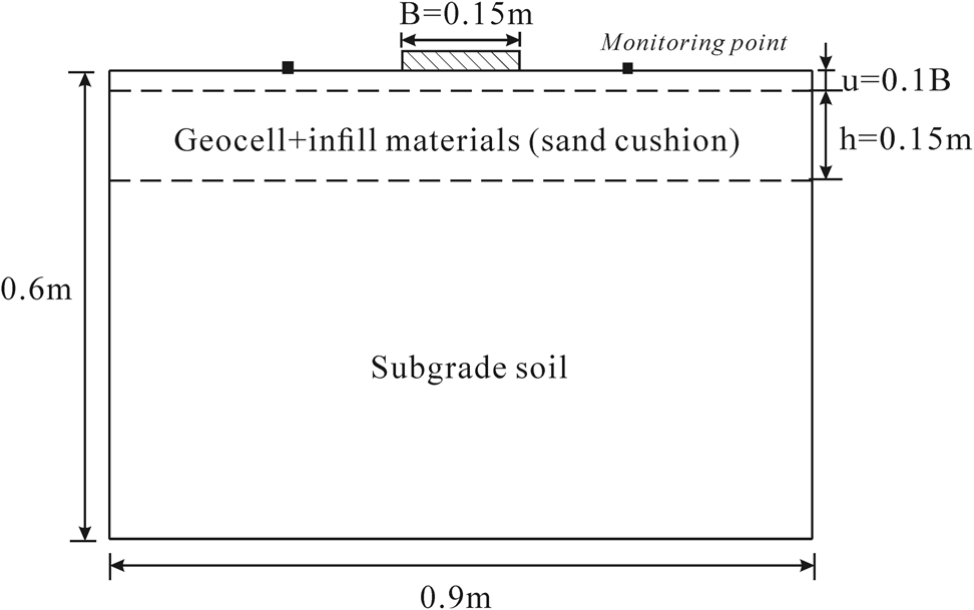
Scheme diagram of numerical simulation.

**Figure 5:**
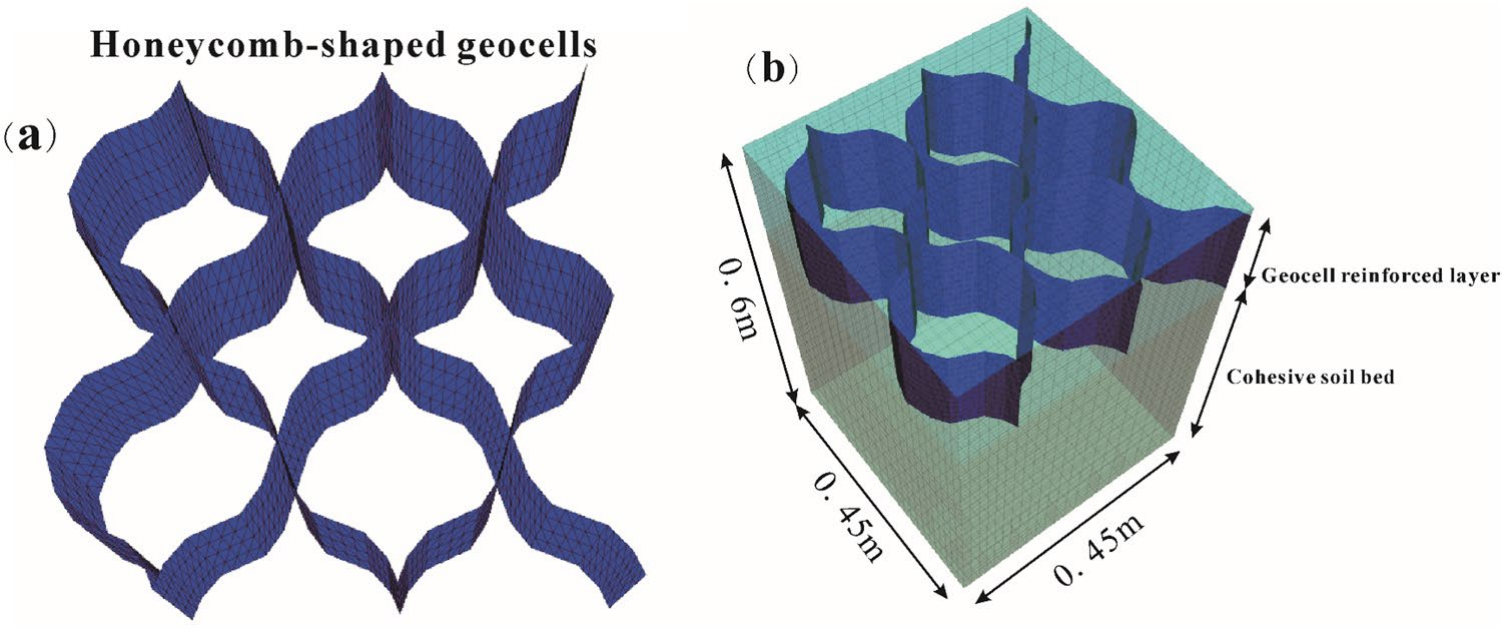
Geometry of FLAC^3D^ model for analysis: (**a**) model of geocell structure; (**b**) model of geocell-reinforced cohesive soil bed.

The fundamental physical properties of the cohesive soil and the particle size distribution are presented in Table 3 and Fig. 3, respectively. Based on these properties, the soil was classified as a low-liquid-limit clay. Furthermore, soil properties subjected to multiple freeze–thaw cycles were considered to simulate long-term reinforced engineering. The testing results of Lu et al.^50^ demonstrated that the soil properties subjected to the freeze–thaw cycle with water reply were closer to the actual engineering in the seasonally frozen soil area. Therefore, the soil samples with the initial compaction of 95% and the water content of 20.2% (the optimum water content) subjected to ten freeze–thaw cycles were used in the unconsolidated undrained triaxial compression tests to obtain the elastic modulus (initial tangent modulus), cohesion, and friction used in numerical calculations. The detailed tests and results were shown in Lu et al.^50^, Xian et al.^51^. The triaxial tests were conducted under three different confining pressures: 50 kPa, 100 kPa, and 150 kPa, with a strain rate of 0.5%/min. The elastic modulus was determined from the stress–strain curve corresponding to a confining pressure of 50 kPa to accurately reflect the low lateral pressure conditions typically encountered in reinforced structures at the site and to ensure improved numerical simulations. Regarding sand or aggregate used in this study, the mechanical parameters were referred to the studies of Hegde and Sitharam^52^. However, the dilation was not shown in their paper. Therefore, in the present study, the dilation angle was taken as 2/3 of friction as suggested by the earlier researchers for similar studies using FLAC^3D^^24,53,54^. For geocells, high-density polyethylene honeycomb-shaped geocell was simulated by the geogrid element (linear elastic constitutive) in this numerical study. The geocell-reinforced layer using the following combination of parameters, $u/B = 0.1$, $d/B = 1.14$, $b/B = 6.0$, $h/B = 1.0$. Geocell modulus was adopted 200 MPa by referencing the numerical study of Yang et al.^44^. Detailed properties of the soils and geocells used in the numerical simulations are represented in Table 4. The schematic diagram and the quarter symmetrical geometry numerical model are shown in Figs. 4 and 5, respectively.

**Table 3:** Basic physical properties of cohesive soil^50^.

Parameters	Value
Liquid limit (%)	38.4
Plastic limit (%)	23.5
Plastic index	14.9
Maximum dry density (kg/m^3^)	1650
Optimum moisture content (%)	20.2
Specific gravity	2.71

**Table 4:** Properties of geocell and different infill materials in numerical modeling.

Parameters	Value
Cohesive soil	
Young modulus, $M_{cs}$ (Pa)	1.26e6
Poisson’s ratio, $\vartheta$	0.3
Cohesion, ${\text{C}}_{cs}$ (Pa)	9.23e3
Friction, $\upvarphi_{cs}$ (°)	8.5
Density, $\uprho$ (kg/m^3^)	2017
Sand	
Young modulus, $M_{s}$ (Pa)	7.5e6
Poisson’s ratio, $\vartheta$	0.3
Cohesion, $c_{s}$ (Pa)	0
Friction, $\upvarphi_{s}$ (°)	35
Dilation, ${\Psi }_{s}$ (°)	23.3
Density, $\uprho$ (kg/m^3^)	1900
Aggregate	
Young modulus, $M_{a}$ (Pa)	8.6e6
Poisson’s ratio, $\vartheta$	0.3
Cohesion, ${\text{c}}_{a}$ (Pa)	0
Friction, $\upvarphi_{a}$ (°)	40
Dilation, ${\Psi }_{a}$ (°)	26.7
Density, $\uprho$ (kg/m^3^)	1950
Geocells	
Cell size (m)	0.225 × 0.18
Equivalent pocket diameter, $d$ (m)	0.171
Geocell height, $h$ (m)	0.15
Thickness of geocell (m)	0.0015
Density $\uprho$ (kg/m^3^)	960
Young modulus, $M_{g}$ (Pa)	200e6
Poisson’s ratio, $\vartheta$	0.45
Interface shear modulus $k_{i}$ (Pa/m)	19.7e6
Interface cohesion, $c_{i}$ (Pa)	$0.8 \times {\text{c}}$
Interface friction, $\upvarphi_{i}$ (°)	${\text{atan }}\left( {0.8 \times \tan \left( {{\upvarphi }} \right)} \right)$

## Results and discussions

In this section, the pressure-settlement curves, the bearing capacity improvement factor ($I_{f}$), the percentage reduction in settlement (PRS), and the surface deformation were used to analyze geocell-reinforced performance with different materials.

### Load bearing capacity and vertical stress distribution

**Figure 6:**
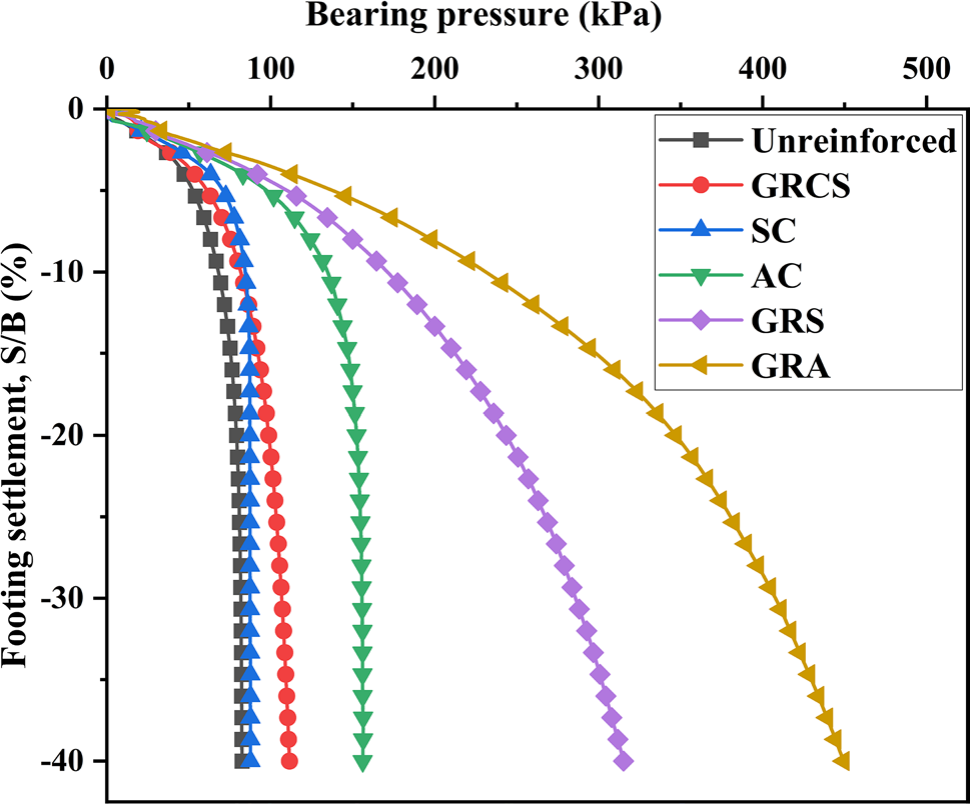
Pressure vs. settlement curves under different cases.

Figure 6 shows the pressure-settlement curves for different reinforced cases. The bearing capacity of geocell-reinforced beds, regardless of the infill material employed, is observed to surpass that of the unreinforced beds, highlighting the efficacy of geocell reinforcement. The pressure-settlement responses from the GRCS, GRS, and GRA curves demonstrate that the soil infill of geocell pockets significantly influences the bearing capacity and the reinforced performance. The case of GRA shows the highest bearing capacity, the GRS case is the second, and the GRCS case is the third. However, the results underscore that cohesive soils provide relatively minimal improvement in terms of bearing capacity. Hegde and Sitharam^35^ claimed that the Indian red soil could increase the load-carrying capacity ten times and decrease the settlement by 70%. The limited improvement in geocell-reinforced performance observed in this study may be attributed to the low modulus and cohesion of the soil. Specifically, the weak mechanical properties of the cohesive soil, such as its low modulus and cohesion, contribute to the relatively modest enhancement in bearing capacity achieved through geocell reinforcement. Regarding the SC and AC cases, the bearing capacity of the GRCS case falls between the two cases. This suggests that a sand/aggregate cushion is more effective than using foundation soil as infill material in geocell-reinforced methods, particularly under certain conditions. It is important to note that geocell-reinforced soil exhibits weaker bearing capacity than aggregate cushion when the infill material is characterized by low shear strength. Moreover, when comparing the GRS case with the SC case (or the GRA case with the AC case), the influence of the modulus and friction of the sand becomes evident in the reinforcement process. It is crucial to avoid overemphasizing the exceptional performance of geocell reinforcement while neglecting the significant role played by the choice of soil infill.

**Figure 7:**
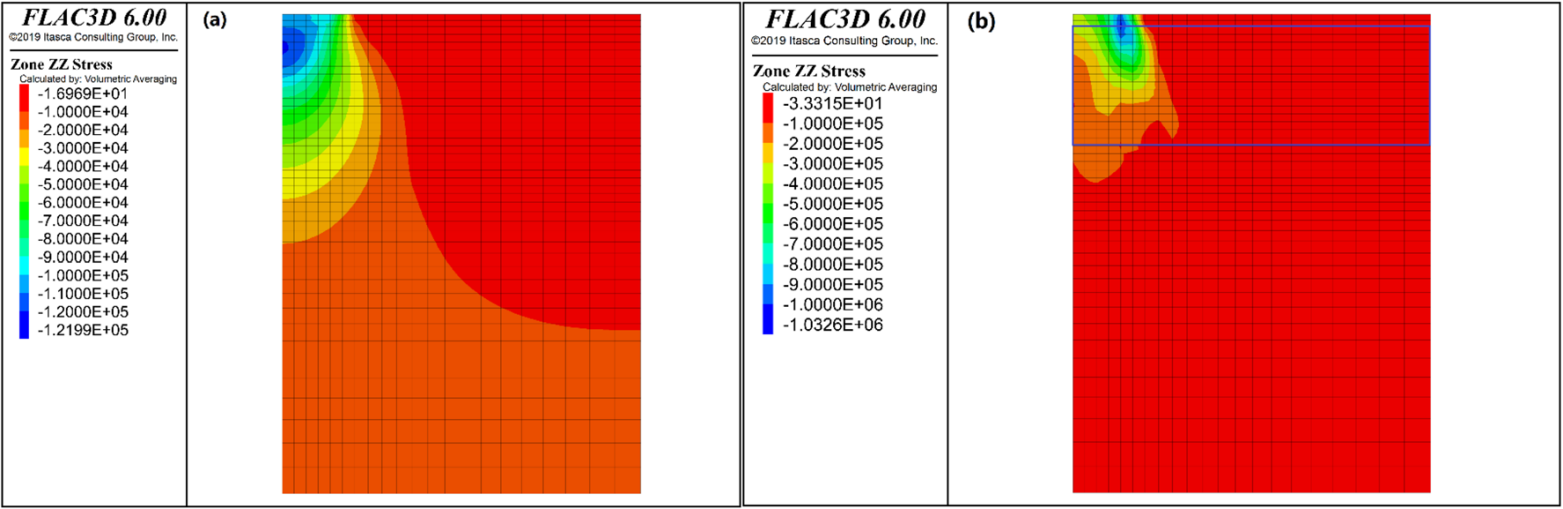
The vertical stress contours of unreinforced and GRA cases: (**a**) Case of unreinforced; (**b**) Case of GRA.

Figure 7 displays the contour plots depicting the distribution of vertical stresses beneath the footing for both the unreinforced and GRA cases. These stress contours correspond to a footing settlement of 40% of the footing width (S/B). In the unreinforced bed, a uniform distribution of vertical stresses is observed extending to a significant depth. However, the vertical stresses are transferred to a shallower depth in the GRA case compared to the unreinforced case. This transfer can be attributed to the lateral confinement the geocell walls provide, which limits the dispersion of stresses. Similar types of observations were also made by Hegde and Sitharam^16^. In addition, Gedela and Karpurapu^20^ claimed that the significant pressure bulb extends from 1.5 times to 2.5 times the footing width below the loading area and on either side, respectively. Also, only little vertical stress contours are noticed to reach the bottom face of the model, indicating that the boundary has few influences on the results.

### Bearing capacity improvement factor

Tafreshi and Dawson^55^, and Dash et al.^13^, etc., used the bearing capacity improvement factor ($I_{f}$) to assess the improvement in bearing capacity with geocell or sand/aggregate cushion reinforcement. $I_{f}$ is a non-dimensional parameter, which is defined as,

1 $$I_{f} = \frac{{q_{r} }}{{q_{0} }}$$

where $q_{r}$ and $q_{0}$ are the bearing capacity of the geocell reinforced and unreinforced soil beds at a given settlement, respectively. Detailed explanation of $I_{f}$ was described by Tafreshi and Dawson^55^. In this study, $\it S/B$S/B was selected at 5%, 10%, 20%, 25%, 30%, and 35% to calculate the value of $I_{f}$.

**Figure 8:**
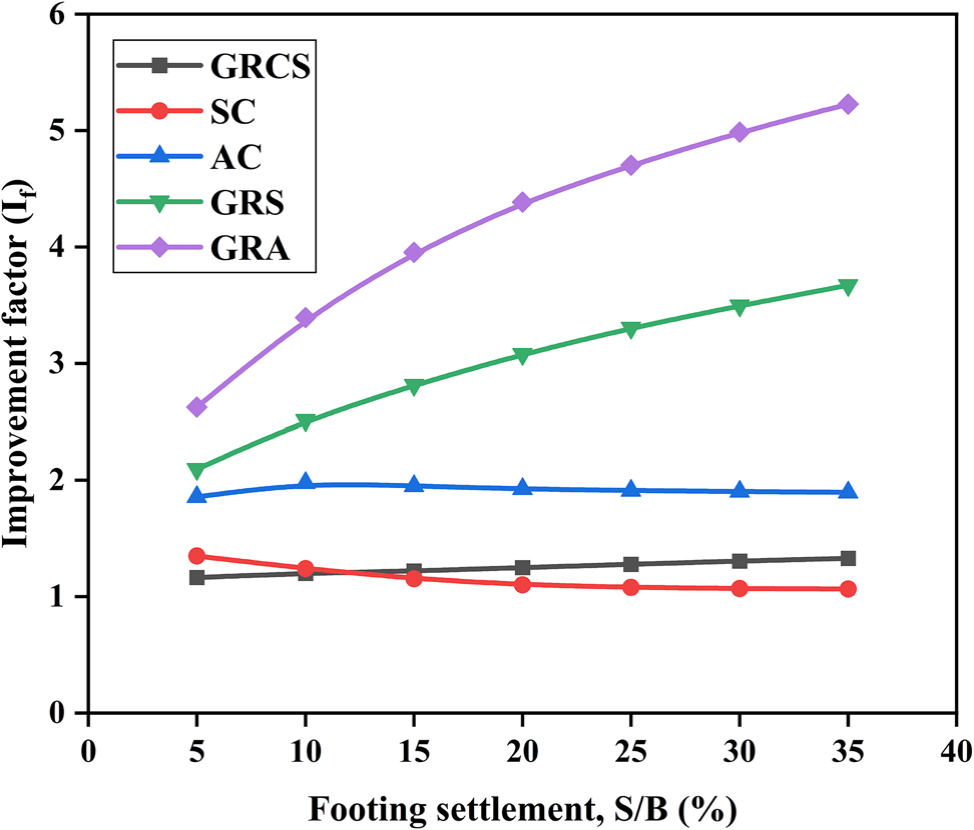
Variation of bearing capacity improvement factors with footing settlement.

Figure 8 illustrates the variation of the bearing capacity improvement factor ($I_{f}$) with respect to the footing settlement. The results indicate that in geocell-reinforced cases, the $I_{f}$ values increase as the footing settlement increases, indicating that the magnitude of the footing settlement strongly influences the reinforced performance. Moreover, by comparing the GRA and AC cases (or the GRS and SC cases), the importance of geocell reinforcement becomes more prominent with higher levels of footing settlement. For instance, in the GRA case, compared to the AC case, the $I_{f}$ value increases by 41.3% when the footing settlement to footing width ratio (S/B) is 5%, and it rises by 175.6% when the S/B is 35%. Geocells primarily enhance bearing capacity when their walls restrict lateral displacement. As a result, when the vertical footing settlement is significant, the soil infill and geocells tend to expand in the lateral direction, leading to increased circumferential deformation and improved geocell-reinforced performance. In essence, the mobilized deformation in the geocell walls enables significant mechanisms of geocell reinforcement.

The infill materials used in geocell reinforcement significantly impact the overall performance. Different infill materials can alter the reinforced performance of soil beds. In cases where cohesionless soil cushions are used (SC and AC cases), the contribution to the soil beds remains consistent regardless of the footing settlement. This distinguishes geocells from other treatment methods. Therefore, when designing geocell reinforcement, it is crucial to consider the mechanical properties of the chosen infill material.

### Percentage reduction in settlement

**Figure 9:**
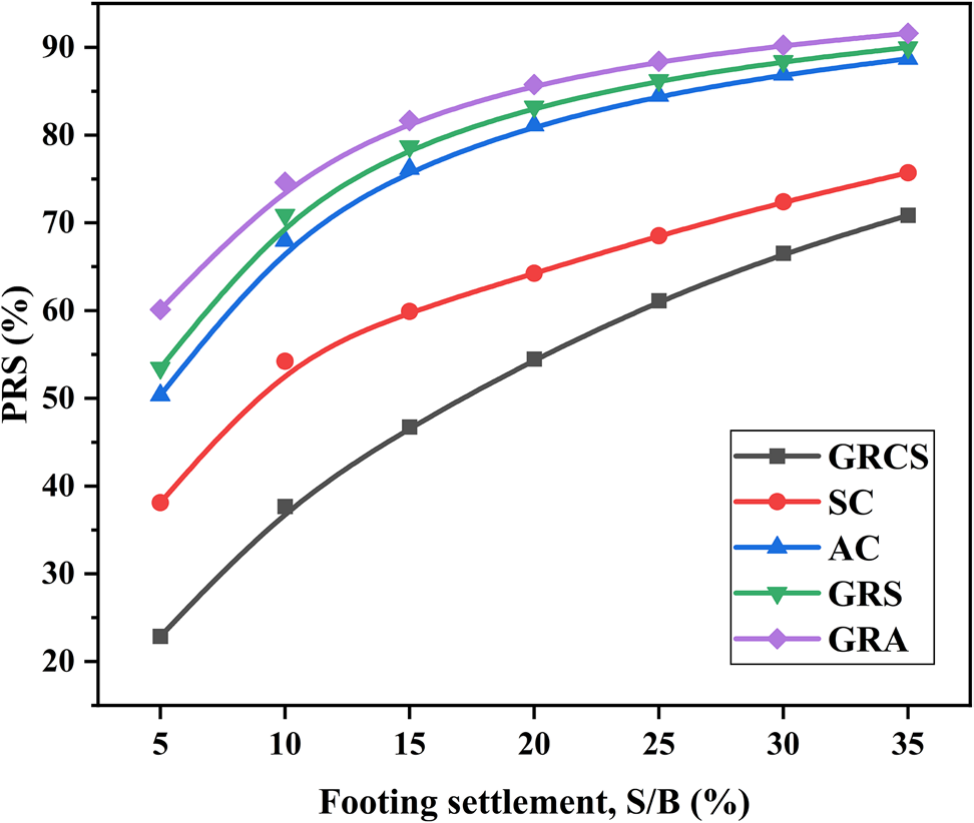
Variation of PRS with footing settlement.

PRS is a non-dimensional parameter that illustrates the performance of geocell-reinforced beds^55^. PRS is defined as follows,

2 $${\text{PRS}} = \left( {\frac{{S_{0} - S_{r} }}{{S_{0} }}} \right) \times 100$$

where $S_{r}$ is the settlement of geocell-reinforced bed at a given bearing pressure corresponding to $S_{0}$ (the settlement of unreinforced bed). Figure 9 shows the variation of the value of PRS with footing settlement. The PRS values increase nonlinearly with the increase of footing settlement and tend to be stable for all reinforcement cases. In terms of PRS values, the reinforced performance of the GRCS case is the weakest, the SC and AC cases are at the position of moderate, and the GRA and GRS show the best-reinforced performance.

The AC and GRA cases also yield similar PRS values, indicating that merely employing a cohesionless soil cushion without geocell reinforcement can significantly reduce settlement. However, this response is primarily observed under conditions of small footing settlement. As discussed in Section “Bearing capacity improvement factor”, sufficient mobilized deformation in geocell walls is critical in promoting the LL effect. In this section, even when the footing settlement exceeds 35%, the corresponding footing pressure for the unreinforced case remains around 100 kPa, insufficient to induce significant lateral displacement in geocells. Therefore, in this scenario, the soil infill in the geocell pockets, rather than the geocells themselves, primarily contributes to the reinforced performance. Furthermore, it is noteworthy that at an S/B ratio of 10%, the GRA case increases by 9.8% compared to the AC case, whereas the GRS case shows a 30.7% growth rate compared to the SC case. This discrepancy suggests that the weaker mechanical properties of cohesionless soil can make the geocell play a much more significant role in the PRS values.

### Surface deformation

**Figure 10:**
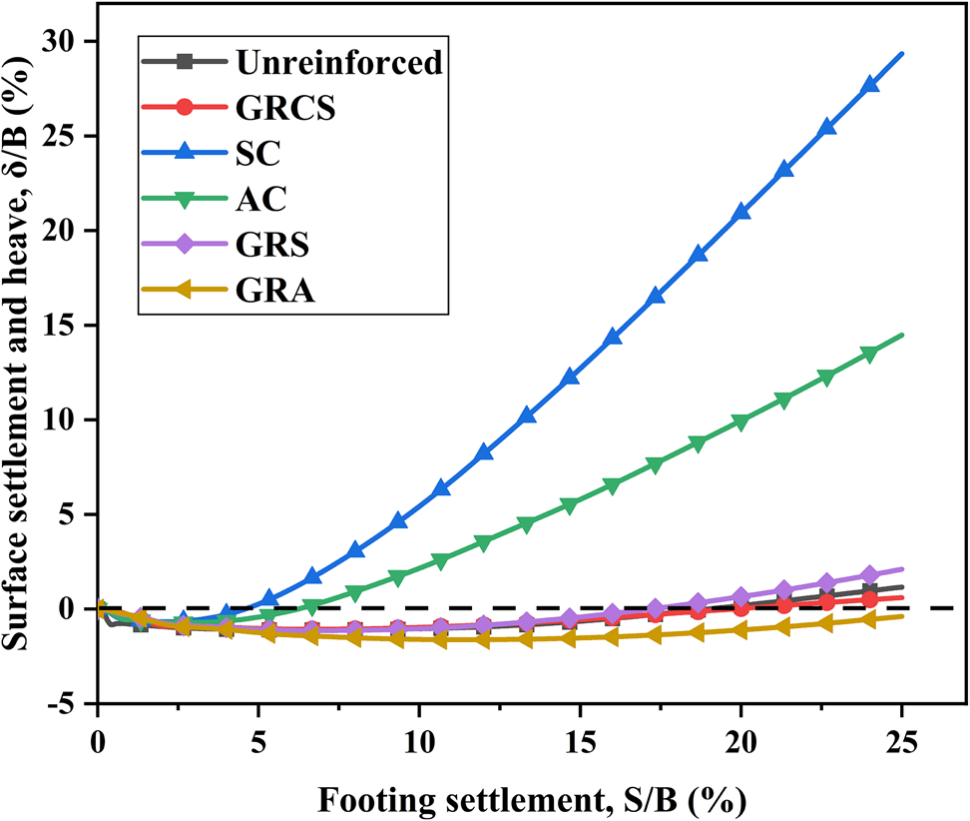
Variation of surface settlement with footing settlement.

**Figure 11:**
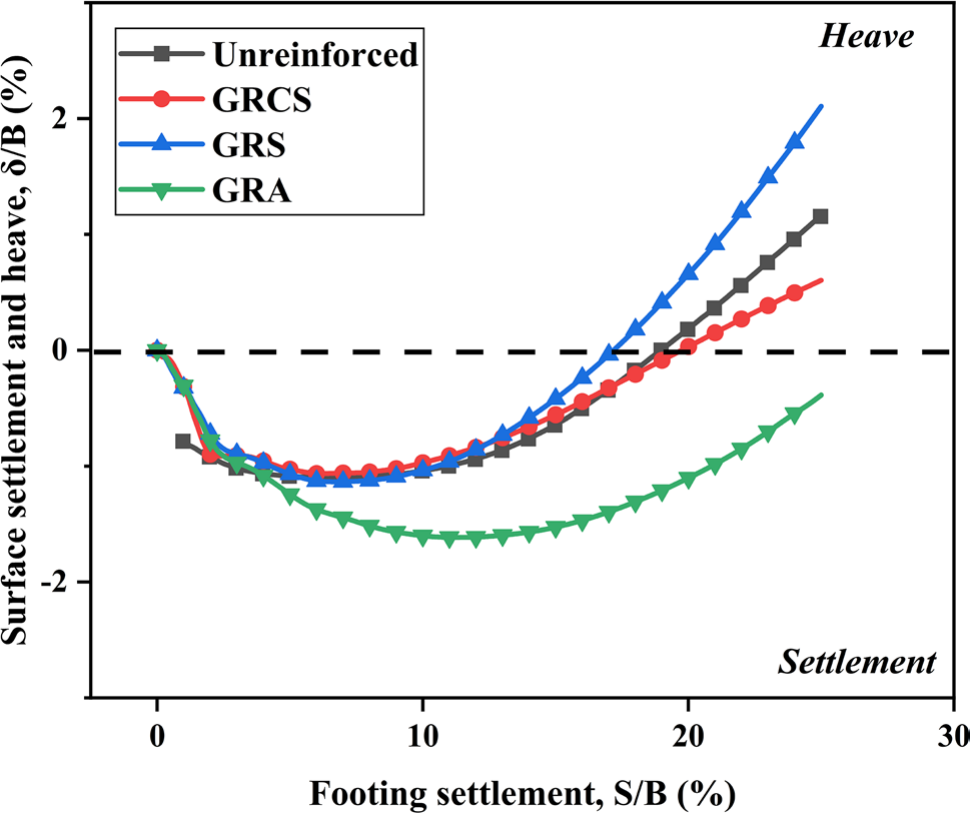
The detailed variation of surface settlement with footing settlement.

Many experimental results demonstrated that the surface around the footing of the unreinforced bed could uplift while the geocells may restrain this behavior^23^. The monitor point at the surface around the footing is illustrated in Fig. 4. Figure 10 presents the variation of surface deformation with footing settlement for different cases. It is observed that there is an evident surface uplift around the footing for the SC and AC cases, as compared to the response of the unreinforced case. This can be attributed to the lack of cohesion in sand or aggregate, a critical factor contributing to significant heave in the sand cushion. However, the presence of geocells reduces the magnitude of heaving, as depicted in Fig. 11. In the unreinforced soil bed, surface heaving is observed, while the presence of geocells in the GRCS and GRA cases helps to restrain surface heaving. Interestingly, in the case of GRS, the innate nature of sand itself, which is more prone to surface heaving, may contribute to the larger heaving observed compared to the response of the unreinforced cases. Additionally, sand's lower mechanical properties than aggregates also play a significant role. Furthermore, lower compaction in geocell-reinforced cases with cohesionless soils as infill materials can also result in surface heaving.

## Parametric study

### Scheme of parametric study

The parametric study investigates the influence of soil infill's mechanical properties on the performance of geocell-reinforced cohesive soil beds. The calculated model presented in Section “Numerical analysis of geocell-reinforced cohesive soil beds” was the baseline model for the follow-up parametric studies. Only one parameter was regarded as the variable while the others were the constants, investigating the effect of the one-parameter on reinforced performance in terms of the $I_{f}$ and PRS values of $S/B = 10\%$. Due to sand and aggregate belonging to the cohesionless soil, only the sand (sand cushion and sand as infill materials) was selected in this section. The specific scheme of the parametric study is presented in Table 5. According to studying the effect of foundation soil (cohesive soil) modulus and cohesion on the reinforced performance, the suitability of the foundation soil (cohesive soil) as the infill materials are discussed. Also, it can be concluded which one (sand or geocell) is the primary factor influencing the reinforced performance from studying the influence of sand modulus and friction on reinforced performance.

**Table 5:** Details of parametric study.

Test series	Details	Variable parameter	Constant parameters
0	Baseline model	None	$M_{g} = 200{ }\,{\text{MPa}}$ u/B = 0.1 h/B = 1 b/B = 6 d/B = 1.14 $M_{cs} = 1.26{ }\,{\text{MPa}}$, ${{\upvarphi }}_{cs} = 8.5$, ${\text{c}}_{cs} = 9.23{ }\,{\text{kPa}}$ ${\text{M}}_{s} = 7.5 \,{\text{MPa}}$, ${{\upvarphi }}_{s} = 35$,${\text{c}}_{s} = 0\,{\text{kPa}}$
1	Effect of foundation soil modulus	$M_{cs} = 0.5{ }1{ }2{ }3{ }4{ }\,{\text{MPa}}$	$M_{g} = 200{ }\,{\text{MPa}}$ u/B = 0.1 h/B = 1 b/B = 6 d/B = 1.14 the other mechanical parameters of cohesive soil and sand
2	Effect of foundation soil cohesion	${\text{c}}_{cs} = 5{ }10{ }20{ }40\,{\text{kPa}}$	
3	Effect of sand modulus	${\text{M}}_{s} = 5 10 20 40 80\,\,{\text{MPa}}$	
4	Effect of sand friction	$\upvarphi_{s} = 20 30 40 50 60 ^\circ$	

### Effect of foundation soil modulus

**Figure 12:**
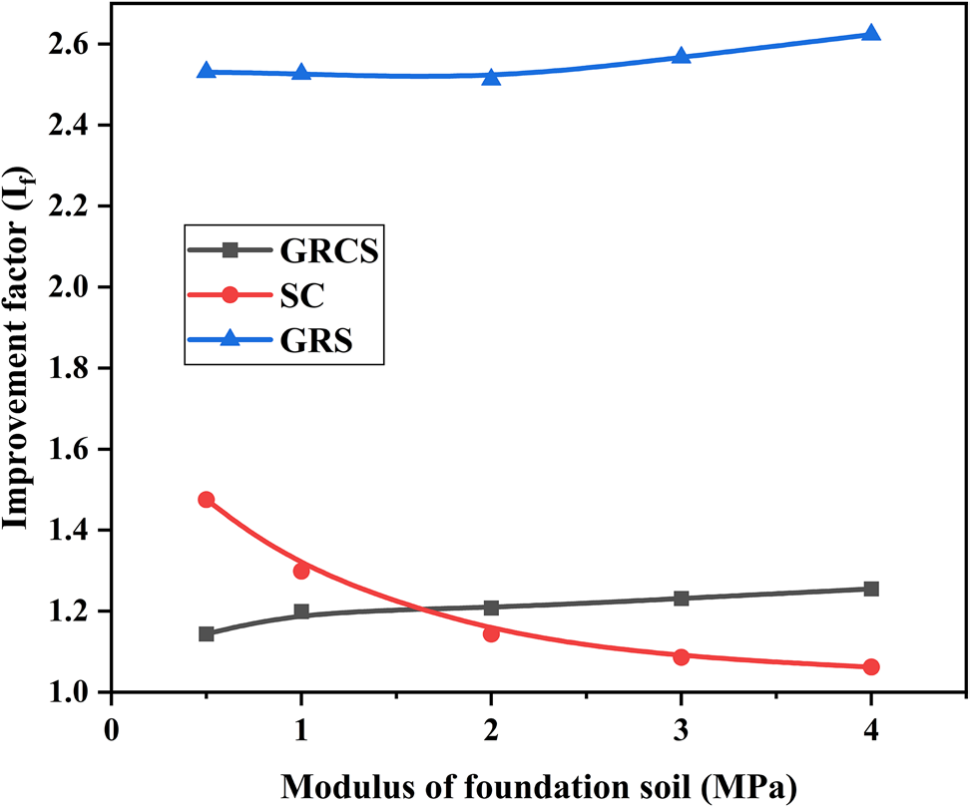
Variation of ${\text{I}}_{f}$ with the modulus of the foundation soil.

**Figure 13:**
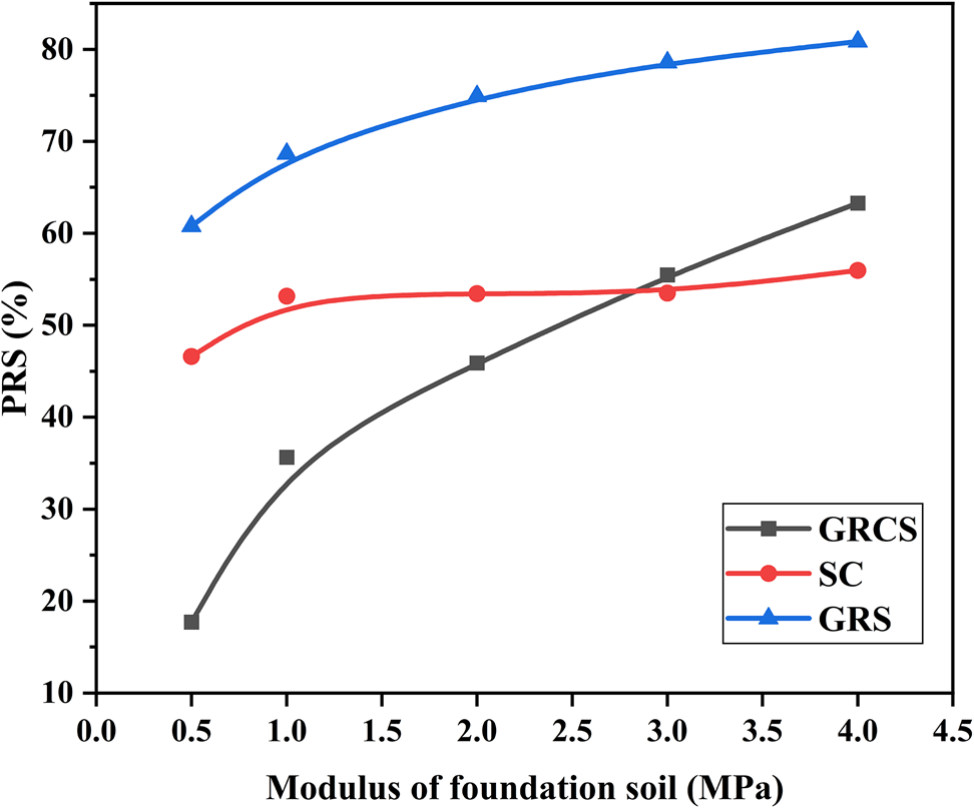
Variation of PRS with the modulus of the foundation soil.

In the present study, the modulus of cohesive soil infill changed with the foundation soils, simulating using the same local foundation soil to fill the geocell pockets. In addition, the $I_{f}$ and PRS values were determined based on the unreinforced case, in which the modulus changed, as Table 5 shown, instead of the modulus of the baseline model. The modulus of foundation soil was varied to 0.5 MPa, 1 MPa, 2 MPa, 3 MPa, and 4 MPa. Figures 12 and 13 show variations of ${\text{I}}_{f}$ and PRS with the foundation soil modulus. In Fig. 12, the ${\text{I}}_{f}$ values increase slightly, even almost keep horizontal, with the increase of foundation soil modulus regardless of whatever infill materials. However, it decreases obviously and tends to be stable with the change of modulus for the SC case. The increase of foundation soil modulus makes the difference between foundation soil and sand cushion gradually close, which leads to the decrease of ${\text{I}}_{f}$. In Fig. 13, the numerical results for PRS align with the abovementioned description. The values of PRS almost keep constant for the SC case, while the values of GRCS and GRS cases increase gradually. Also, the growth of the GRCS case is larger than the GRS case due to the foundation soil modulus increasing. Combined with Figs. 12 and 13, using the foundation soils as the infill materials for the geocell-reinforced cohesive soil bed contributes little to bearing capacity but benefits to decrease the footing settlement.

### Effect of foundation soil cohesion

**Figure 14:**
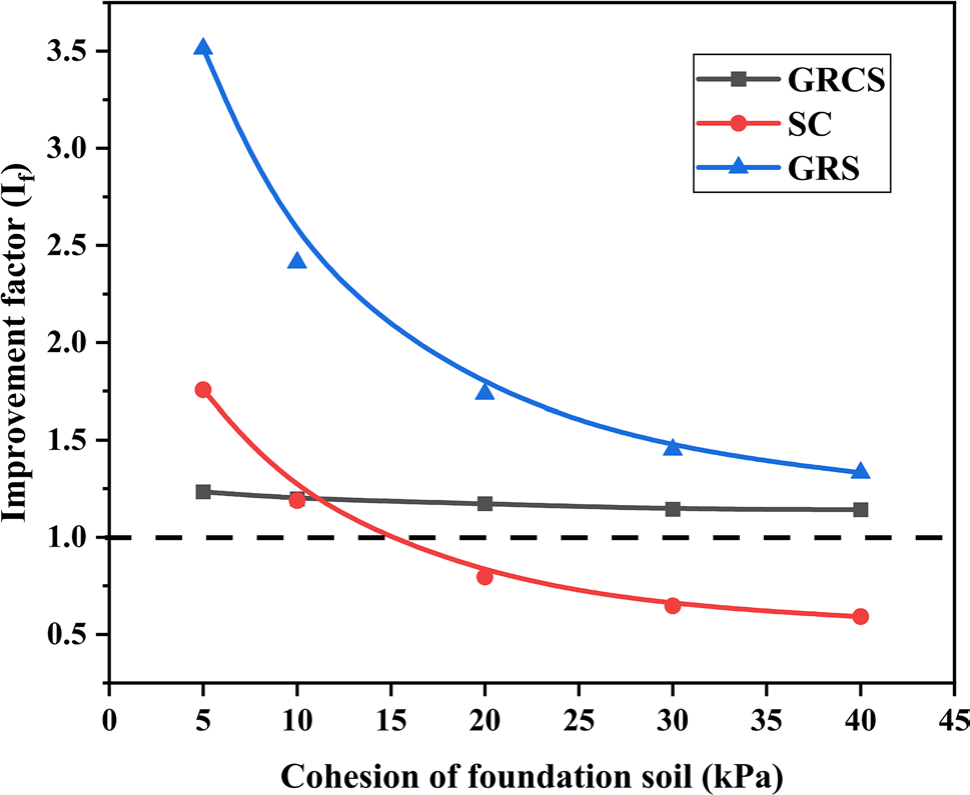
Variation of ${\text{I}}_{f}$ with the cohesion of foundation soil.

**Figure 15:**
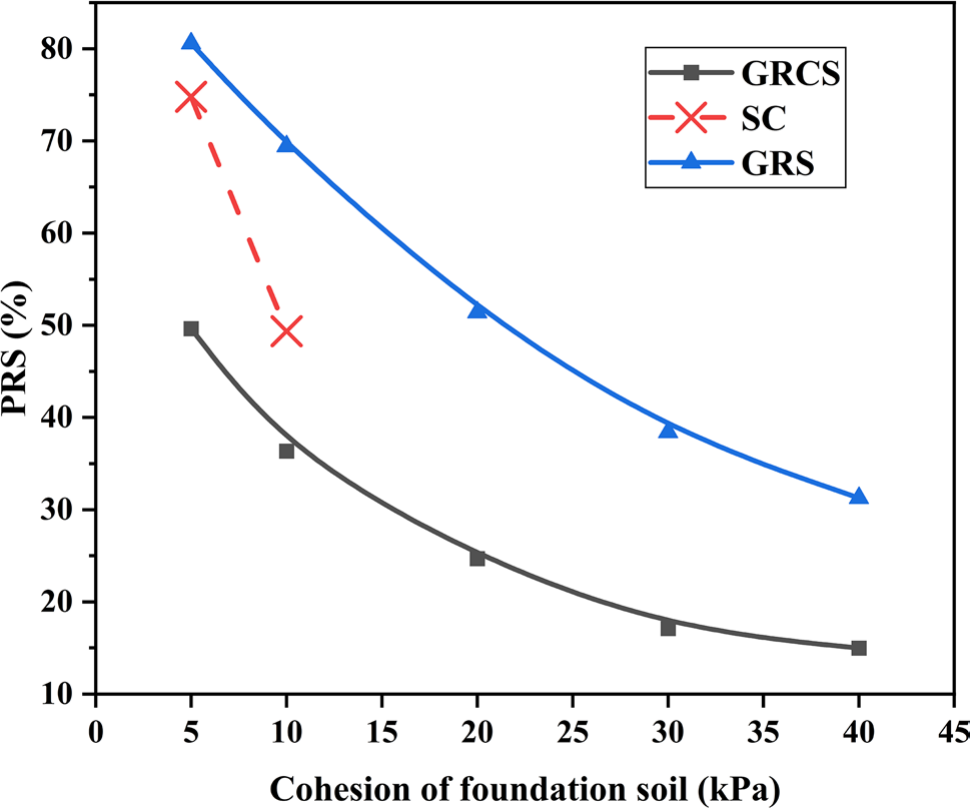
Variation of PRS with the cohesion of foundation soil.

Figures 14 and 15 show the variation of ${\text{I}}_{f}$ and PRS with the foundation soil cohesion, respectively. In this numerical study, the interface cohesion of the geocell was changed with the cohesion of the foundation soil for the GRCS case. In Figs. 14 and 15, ${\text{I}}_{f}$ and PRS values of SC and GRS cases decrease parallelly with the increase of foundation soil modulus, indicating the geocell-reinforced layer and sand cushion benefits little to the high bearing capacity foundation or subgrade. The increasing of foundation soil cohesion contributes to the increase of bearing capacity, which causes the ${\text{I}}_{f}$ and PRS values decrease due to the mechanical properties of geocell and sand keeping the same. In terms of the case of GRCS, the values decrease slightly and almost keep linear, suggesting the soil cohesion has little improvement on the geocell-reinforced cohesive soil beds. It is noted that, for three points in Fig. 14, the value of ${\text{I}}_{f}$ of SC case is less than zero, demonstrating that using the sand cushion to treat the soil beds is unnecessary for the cohesive soil beds with high bearing capacity. That is why there is no continuous curve of the PRS value for the SC case in Fig. 15.

Figures 12, 13, 14, 15 demonstrate that foundation soils can be utilized as infill materials. However, this approach provides limited improvement to bearing capacity while reducing footing settlement. Therefore, in the case of GRCS, the most suitable infill material is cohesive soil with a higher modulus and lower cohesion. Additionally, it is possible that cohesive soil may not be the optimal choice for infill material when compared to cohesionless soil.

### Effect of sand modulus

**Figure 16:**
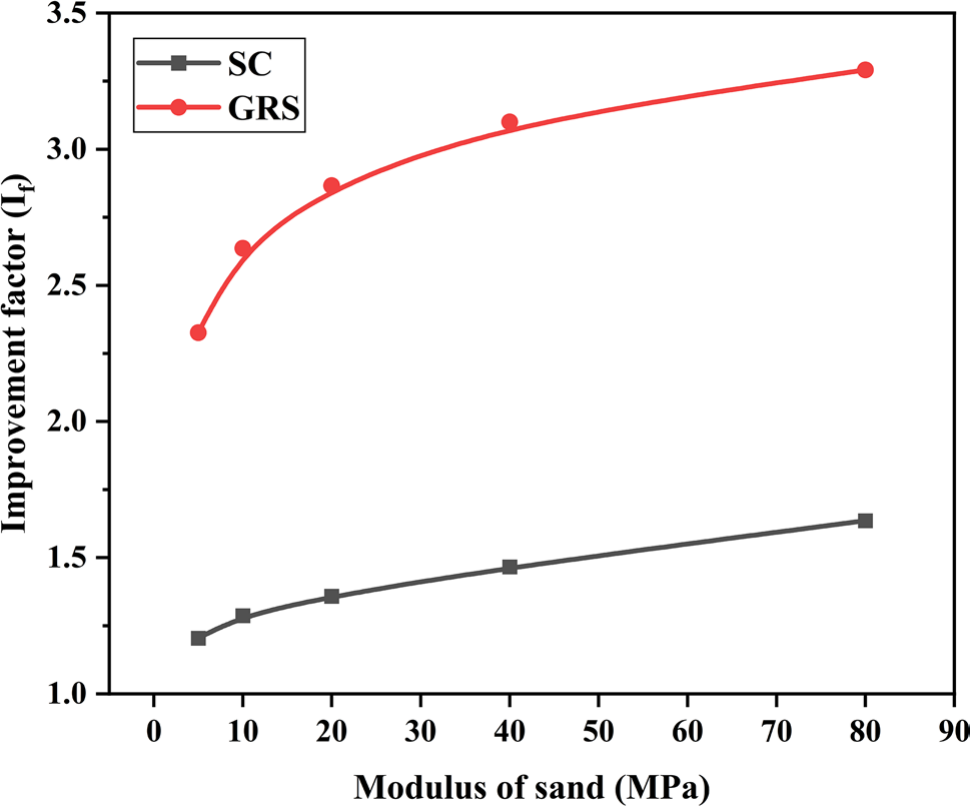
Variation of ${\text{I}}_{f}$ with the modulus of sand.

**Figure 17:**
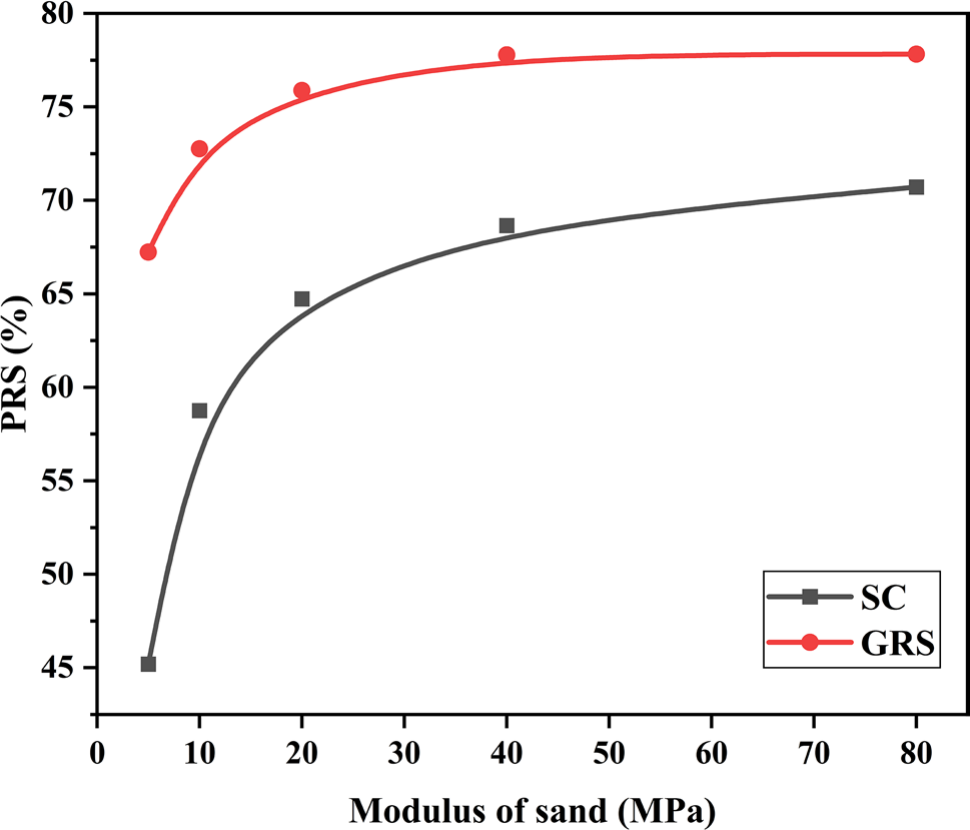
Variation of PRS with the modulus of sand.

This section discusses the impact of different sand moduli on reinforced performance. The GRCS case is not included in the analysis as the mechanical properties of the foundation soil remain unchanged. Figures 16 and 17 present the variations of ${\text{I}}_{f}$ and PRS, respectively, with respect to the sand modulus. In the SC case, the values of ${\text{I}}_{f}$ range from 1.2 to 1.6, indicating that the sand cushion's modulus has minimal influence on the bearing capacity. However, it is worth noting that the sand cushion on the foundation effectively reduces the footing settlement without significantly improving the bearing capacity, as illustrated in Fig. 17. In contrast, for the GRS case, both ${\text{I}}_{f}$ and PRS exhibit a similar pattern of non-linear increase followed by stabilization with increasing sand modulus. Higher infill sand modulus contributes to enhanced bearing capacity and reduced footing settlement.

In sum, the increase of sand modulus benefits the performance of treated cohesive beds, for the SC or the GRS cases. Hence, to distinguish the role of sands themselves and geocells, the percentage of contribution of each one should be calculated. For example, when the sand modulus is 5 MPa, the ${\text{I}}_{f}$ for the SC and GRS cases is 1.20 and 2.33. The value of (1.2–1.0)/(2.33–1.0) = 15.0% represents the contribution of infill materials (sand) in geocell reinforcement for the GRS case. Also, the contribution of geocells is 1–15.0% = 85.0%. This method presents the detailed percentage in Tables 6 and 7. It is observed that the contribution of geocells decreases with an increase in sand modulus. This indicates that a higher modulus of sand can improve the reinforced performance, but it weakens the effectiveness of geocells. Integrating the information from Fig. 16 and 17, it can be concluded that a sand modulus of 20 MPa is the optimal choice.

**Table 6:** Role of sand and geocell in the reinforcement based on ${\text{I}}_{f}$ value.

	5 MPa (%)	10 MPa (%)	20 MPa (%)	40 MPa (%)	80 MPa (%)
Sand	15.0	17.6	19.2	22.2	27.8
Geocell	85.0	82.4	80.8	77.8	72.2

**Table 7:** Role of sand and geocell in the reinforcement based on PRS value.

	5 MPa (%)	10 MPa (%)	20 MPa (%)	40 MPa (%)	80 MPa (%)
Sand	67.2	80.7	85.3	88.3	90.9
Geocell	32.8	19.3	14.7	11.7	9.1

### Effect of sand friction

**Figure 18:**
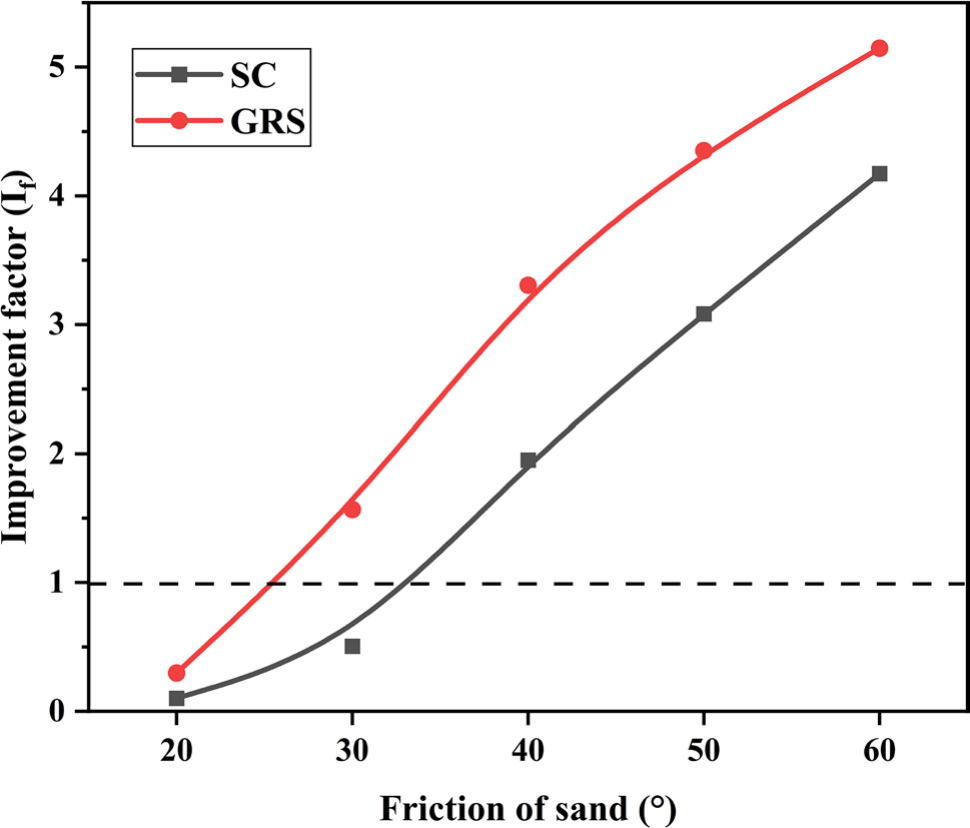
Variation of ${\text{I}}_{f}$ with the friction of sand.

**Figure 19:**
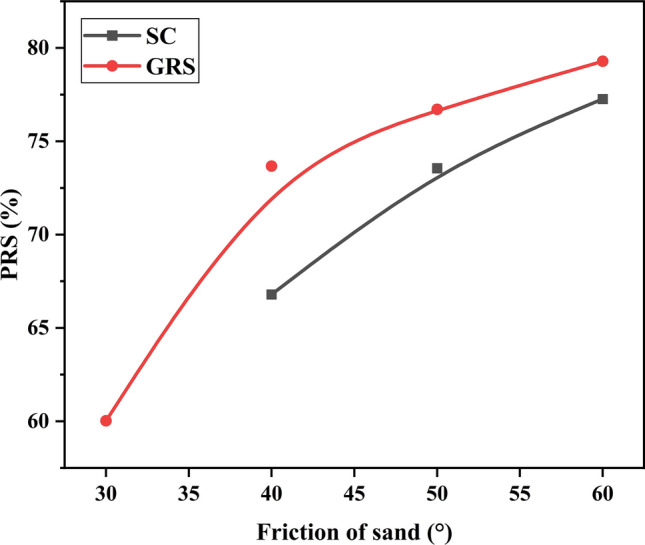
Variation of PRS with the friction of sand.

Figures 18 and 19, respectively, show the variations of ${\text{I}}_{f}$ and PRS with sand friction. Figure 18 demonstrates that three points in the ${\text{I}}_{f}$ values are below 1, indicating that the treatment applied does not enhance the performance of the soil beds. Consequently, three points are missing in Fig. 19 as well. It can be concluded that larger sand friction can linearly improve the bearing capacity and nonlinearly reduce the settling of the footing. In the GRS case, regarding the PRS values, the benefit in terms of settlement gradually stabilizes when the sand friction exceeds 40°. Using the methodology described in Section “Effect of sand modulus”, the contributions of sand and geocells towards reinforcement are calculated separately and detailed results are presented in Tables 8 and 9. Notably, sand plays a critical role in enhancing the bearing capacity and reducing settlement for geocell reinforcement. In contrast, geocells exhibit minimal contribution to the settlement reduction, as seen in Table 9. Generally, the reinforced performance can be enhanced by increasing the sand friction for both the GRS and SC cases. However, as the sand friction increases, the contribution of sand becomes more significant. In this study, a sand friction value of 40° can be considered the optimal choice for infill materials, ensuring well-reinforced performance and promoting the contribution of geocells.

**Table 8:** Role of sand and geocell in the reinforcement based on ${\text{I}}_{f}$ value.

	20	30	40 (%)	50 (%)	60 (%)
Sand	N/A	N/A	41.3	62.2	76.5
Geocell	N/A	N/A	58.7	37.8	23.5

**Table 9:** Role of sand and geocell in the reinforcement based on PRS value.

	20	30	40 (%)	50 (%)	60 (%)
Sand	N/A	N/A	90.7	95.9	97.5
Geocell	N/A	N/A	9.3	4.1	2.5

## Conclusions

This paper presents a series of numerical calculations to study the performance of geocell-reinforced cohesive soil beds with different infill materials. The cohesive soil used in the simulation was taken from a seasonally frozen soil area in China. Some laboratory tests were conducted to obtain some physical and mechanical soil parameters to simulate for considering the long-term service status of subgrades. Initially, a model test conducted by Latha and Somwanshi^24^ was selected to validate the suitability for the FLAC^3D^ by using the structure element. Then, the verified model was extended to the geocell-reinforced cohesive soil bed models. Three cases were analyzed (i.e., geocell reinforcement with foundation soil as infill materials, geocell reinforcement with sand/aggregate as infill materials, and sand/aggregate cushion). Furthermore, parametric studies were used to analyze the influence of soil mechanical properties on the geocell-reinforced performance. According to the numerical results, the following conclusions can be extracted.

1. Five reinforcement and unreinforced cases were analyzed to study the geocell-reinforced performance by considering different infill materials. The numerical bearing capacity results indicate that geocell-reinforced cohesive beds with cohesionless soil as the materials are superior to the reinforcement with infill of cohesive local soils (foundation soil). The mechanical properties of soil infill are the critical factors influencing reinforced performance. Further, by comparing the sand/aggregate cushion, it can be noted that the superior geocell reinforcement should not be overemphasized and neglect the properties of soil infill.
2. Geocells can improve the bearing capacity of cohesive soil beds with sand or aggregate as the infill materials. However, the geocells contribute little to the bearing capacity when using the foundation soil (cohesive soil) as the infill material. In addition, the ${\text{I}}_{f}$ value of the GRA case increases by 41.3% based on the AC case with the $S{/}B$ equaling to 5%, also the value increases by 175.6% with $S{/}B$ equaling to 35%. Hence, geocells benefit more bearing capacity when the geocell walls are mobilized a lot in the lateral direction.
3. While geocells may not significantly improve the bearing capacity when using foundation soils as infill materials, they can still be beneficial in reducing footing settlement, as demonstrated by changes in PRS values. In the GRA case, there is a 9.8% increase in the PRS value compared to the AC case with a reinforcement ratio ($S{/}B$) of 10%. Conversely, the GRS case exhibits a much higher growth rate of 30.7% in PRS value than the SC case. It can be inferred that the weaker mechanical properties of cohesionless soil make the geocell play a more significant role in reducing footing settlement, thus contributing to the greater increment observed in the GRS case compared to the GRA case.
4. Various mechanical parameters influence geocell-reinforced performance. In the case of GRCS, better performance is observed with higher soil modulus and lower cohesion. For the GRS case, optimal results are achieved with a sand modulus of 20 MPa and a friction angle of 40°. These parameters contribute to improved reinforcement performance and maximize the effectiveness of geocells.
5. This study can provide a reference to the designer to select an optimum material to fill the geocells overlaying the cohesive bed and make the researcher understand the mechanism of the influence of infill materials on the reinforced performance. However, the corresponding results were not validated by the experimental results. The following studies should focus on using the model tests for validation.

## Data Availability

The datasets used and/or analyzed during the current study are available from the corresponding author on reasonable request.

## List of symbols

B: Width of footing (m)
b: Width of geocell mattress
$c_{a}$: Cohesion of aggregate (kPa)
$c_{cs}$: Cohesion of cohesive soil (kPa)
$c_{i}$: Interface cohesion (kPa)
$c_{s}$: Cohesion of sand (kPa)
d: Equivalent pocket diameter (m)
h: Geocell height (m)
${\text{k}}_{i}$: Interface shear modulus (MPa/m)
$M_{a}$: Young modulus of aggregate (MPa)
$M_{cs}$: Young modulus of cohesive soil (MPa)
$M_{g}$: Young modulus of geocell (MPa)
$M_{s}$: Young modulus of sand (MPa)
S: Settlement of footing (m)
t: Thickness of geocell (mm)
u: Depth of placement of geocell layer (m)
$\delta$: Surface settlement and heave (mm)
$\vartheta$: Poisson’s ratio (dimensionless)
$$\uprho$$: Density (kg/m^3^)
$\upvarphi_{a}$: Shearing resistance angle of aggregate (°)
$\upvarphi_{cs}$: Shearing resistance angle of cohesive soil (°)
$\upvarphi_{i}$: Interface friction angle (°)
$\upvarphi_{s}$: Shearing resistance angle of sand (°)
${\uppsi }_{a}$: Dilation angle of aggregate (°)
${\uppsi }_{s}$: Dilation angle of sand (°)
